# Correction: Anaphase onset requires CKS-1–mediated destruction of securin in meiosis I and cyclin B1 in meiosis II

**DOI:** 10.1083/jcb.20250208703122026c

**Published:** 2026-03-19

**Authors:** Jie Yang, Eisuke Sumiyoshi, Bruce Bowerman

Vol. 225, No. 2 | https://doi.org/10.1083/jcb.202502087 | January 22, 2026

After publication, the authors realized that they had neglected to cite related papers by Kõivomägi and colleagues (Kõivomägi et al., 2011 and Kõivomägi et al., 2013) and Yu and Reed (Yu and Reed, 2005).

The following articles are now addressed in the text:

Kõivomägi, M., E. Valk, R. Venta, A. Iofik, M. Lepiku, E.R.M. Balog, S.M. Rubin, D.O. Morgan, and M. Loog. 2011. Cascades of multisite phosphorylation control Sic1 destruction at the onset of S phase. *Nature.* 480:128-132. https://doi.org/10.1038/nature10560

Kõivomägi, M., M. Örd, A. Iofik, E. Valk, R. Venta, I. Faustova, R. Kivi, E.R.M. Balog, S.M. Rubin, and M. Loog. 2013. Multisite phosphorylation networks as signal processors for Cdk1. *Nat. Struct. Mol. Bio.* 20:1415-1424. https://doi.org/10.1038/nsmb.2706

Yu, V.P.C.C., and S.I. Reed. 2005. Cks1 id dispensable for survival in *Saccharomyces cerevisiae*. *Cell Cycle.* 3:1402-1404. https://doi.org/10.4161/cc.3.11.1208

In the Discussion section, the second paragraph of the subsection titled “Meiosis II anaphase onset in *C. elegans* oocytes requires CYB-3 presence and CYB-1 destruction” has been edited. A citation for Kõivomägi et al. (2013) was added to the first sentence. The last sentence of this paragraph was changed from “However, while mutational inactivation of the CKS anion pocket in budding yeast altered the phosphorylation status of some CDK-1 targets, loss of the anion pocket was not lethal, in contrast to the lethality caused by complete loss of budding yeast CKS (McGrath et al., 2013)” to “However, while mutational inactivation of the CKS anion pocket in budding yeast altered the phosphorylation status of some CDK-1 targets, loss of the anion pocket was not lethal (Kõivomägi et al., 2011), and indeed null mutations in budding yeast CKS result in slower growth and misshapen cells but are not lethal (Yu and Reed, 2005).” In the paragraph below, the additions are indicated in bold, and deletions are crossed out.

**Meiosis II anaphase onset in *C. elegans* oocytes requires CYB-3 presence and CYB-1 destruction (**second paragraph**)**

“First, in budding yeast, the anion pocket was shown to be required for CKS to bind phosphorylated CDK targets and promote their destruction through further phosphorylation by CDK-1 (Asfaha et al., 2022; **Kõivomägi et al., 2013;** McGrath et al., 2013). Presumably, the increased phosphorylation either promotes target association with the APC/C adaptor Cdc20 or otherwise makes targets more suitable for poly-ubiquitylation. However, while mutational inactivation of the CKS anion pocket in budding yeast altered the phosphorylation status of some CDK-1 targets, loss of the anion pocket was not lethal, in contrast to the lethality caused by complete loss of budding yeast CKS (McGrath et al., 2013)** (Kõivomägi et al., 2011), and indeed null mutations in budding yeast CKS result in slower growth and misshapen cells but are not lethal (Yu and Reed, 2005)**.”

The conclusions of the paper are not affected by this error, and all discussion of the data presented remains correct. This error appears in print and in PDFs downloaded on or before March 16, 2026. The authors apologize for any confusion this may have caused.
